# Dietary intervention with a specific micronutrient combination for the treatment of patients with cardiac arrhythmias: the impact on insulin resistance and left ventricular function

**DOI:** 10.1186/s12872-018-0954-6

**Published:** 2018-12-03

**Authors:** Elke Parsi, Norman Bitterlich, Anne Winkelmann, Daniela Rösler, Christine Metzner

**Affiliations:** 1 Outpatient Practice of Cardiology, Suermondtstr. 13, D-13053 Berlin, Germany; 2 Medicine and Service Ltd, Department of Biostatistics, Boettcherstr. 10, D-09117 Chemnitz, Germany; 3 Bonn Education Association for Dietetics r. A, Fuerst-Pueckler-Str. 44, D-50935 Cologne, Germany; 4 Department of Internal Medicine III, Uniklinik RWTH Aachen, Pauwelsstraße 44, D-52074 Aachen, Germany

**Keywords:** Diastolic LV function, LVMI, Dietary intervention, Glucose metabolism, Premature beats

## Abstract

**Background:**

Cardiac arrhythmias (CA) are very common and may occur with or without heart disease. Causes of these disturbances can be components of the metabolic syndrome (MetS) or deficits of micronutrients especially magnesium, potassium, B vitamins and coenzyme Q10. Both causes may also influence each other. Insulin resistance (IR) is a risk factor for diastolic dysfunction. One exploratory outcome of the present pilot study was to assess the impact of a dietary intervention with specific micronutrients on the lowering of IR levels in patients with CA with the goal to improve the left ventricular (LV) function.

**Methods:**

This was a post hoc analysis of the randomized double blind, placebo-controlled pilot study in patients with CA (VPBs, SVPBs, SV tachycardia), which were recruited using data from patients who were 18–75 years of age in an Outpatient Practice of Cardiology. These arrhythmias were assessed by Holter ECG and LV function by standard echocardiography. Glucose metabolism was measured by fasting glucose, fasting insulin level and the Homeostasis Model Assessment of IR (HOMA-IR) at baseline and after 6 weeks of dietary supplementation.

**Results:**

A total of 54 randomized patients with CA received either a specific micronutrient combination or placebo. Dietary intervention led to a significant decrease in fasting insulin ≥58 pmol/l (*p* = 0.020), and HOMA-IR (*p* = 0.053) in the verum group after 6 weeks. At the same time, parameters of LV diastolic function were improved after intervention in the verum group: significant reduction of LV mass index (*p* = 0.003), and in tendency both a decrease of interventricular septal thickness (*p* = 0.053) as well as an increase of E/A ratio (*p* = 0.051). On the other hand, the premature beats (PBs) were unchanged under verum.

**Conclusions:**

In this pilot study, dietary intervention with specific micronutrient combination as add-on to concomitant cardiovascular drug treatment seems to improve cardio metabolic health in patients with CA. Further studies are required.

**Study registration:**

The study was approved by the Freiburg Ethics Commission International and was retrospectively registered with the U.S. National Institutes of Health Clinical Trials gov ID NCT 02652338 on 16 December 2015.

## Background

Supraventricular (SVPBs) and ventricular premature beats (VPBs) are very common arrhythmias in patients with cardiovascular disorders. The incidence of SVPBs in Electrocardiogram (ECG) at rest is present in 10–20% [1], VPBs in 5–10% [2]. The prevalence is increasing with age. Patients with components of MetS like arterial hypertension (AH), abdominal obesity, type 2 diabetes mellitus (T2DM), impaired glucose tolerance (IGT) or HOMA-IR also show in addition to CA in different degree disturbances in cardiac metabolism and LV function. Based on abdominal obesity, IGT driven by IR leads already at an early stage to changes of diastolic function in different degree [3, 4]. The diastolic dysfunction is described with different parameters [5, 6] but according to the current ESC Guidelines for the diagnosis and treatment of acute and chronic heart failure, one of important parameter to describe the diastolic function is the left ventricular mass index (LVMI) [7].

Numerous studies on therapeutic interventions with magnesium [8–12], potassium [13], B vitamins [14–19], and Coenzyme Q10 [20–24] for CA and cardiometabolic risk factors have been published. An insufficient supply of magnesium can be clinical relevant as a trigger of CA and can also be linked with different components of the MetS like T2DM, and insulin resistance as well as by therapy with proton-pump inhibitors [25] and diuretics [12]. It may be caused by poor oral intake, elevated renal loss, diarrhea or alcoholism. The hypomagnesemia is also often combined with hypokalemia [26].

One objective of this pilot study was to investigate, if daily dietary intervention with a specific micronutrient combination improved LV function of CA in patients with disturbances in glucose metabolism after 6 weeks.

## Methods

### Study population

This was a post hoc analysis of the randomized double blind, placebo-controlled pilot study in patients with CA, which were recruited from patients who were 18–75 years of age in an Outpatient Practice of Cardiology in Berlin, Germany. 74 Caucasian patients with cardiac arrhythmias (VPBs, SVPBs, SV tachycardia) were screened from April 2014 to July 2015. Overall, patients who completed the study participated in 5 visits in the Outpatient Practice of Cardiology. At the first visit the patients were informed about the study in detail and the necessary examinations for inclusion and exclusion criteria were carried out. If the criteria were met and the patient gave his written consent to participate in the study, the placebo tablets for the run-in phase were issued at the second visit. Visit 3 marked the time baseline, again the inclusion and exclusion criteria were examined and the patients randomized. A further visit followed after a 3-week intervention phase, before 6 weeks follow-up.

Inclusion criteria were: ≥ 500 VPBs or ≥ 200 SVPBs or ≥ 10 SV tachycardia’s in Holter ECG at least 18 h. The general exclusion criteria were age > 75 years, LVEF ≤40%, intake of spironolactone > 50 mg/d, torasemide > 20 mg/d, supplementation of vitamins and minerals, hypo- or hyperkalemia, hypo- or hypermagnesemia, impaired renal function, hyperthyreosis, cardiac pacemaker, acute or chronic diarrhoea. Fourteen screened patients don’t meet the inclusion criteria: They have not had the amount of supraventricular or ventricular ectopic beats, and one patient had a hypomagnesemia.

**Fig. 1:**
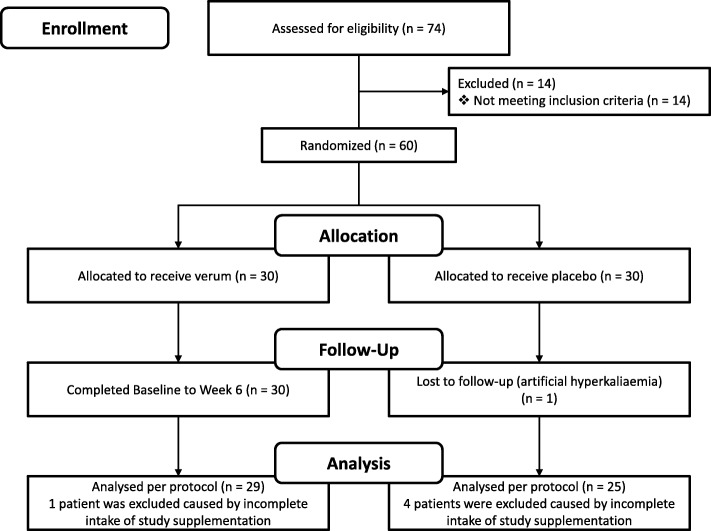
Trial Profile

Figure 1 shows the trial profile of the 74 screened patients. All included subjects received placebo administration (2 tablets 2 times a day) during a one-week run-in period. After evaluation of the Holter ECG at baseline and check of the inclusion criteria 60 patients were randomly assigned to the verum or placebo group. Duration of dietary intervention was 6 weeks. Over this period of the randomized double blind, placebo-controlled pilot study, participants were required to take 2 tablets 2 times a day of verum (5 kcal, 145.8 mg magnesium, 469.2 mg potassium, 3.0 μg Vitamin B12, 400.0 μg folic acid, 48.0 mg niacin and 60.0 mg coenzyme Q10) or placebo (microcrystalline cellulose) with 200 ml water.

### Outcomes for glucose metabolism and LV function

The exploratory outcomes were the changes in HOMA-IR and LV function after 6 weeks of dietary intervention in patients with CA.

### Clinical measurements of components of the metabolic syndrome

At baseline the waist circumference (WC) was determined with a flexible tape with a measuring deviation nearest to 0.5 cm. WC was measured at the level midway between the lower rib margin and the iliac crest after breathing out normally and in standing position. MetS was determined by using diagnostic criteria of the International Diabetes Federation (IDF) [27]. The IDF definition requires increased WC, namely ≥94 cm and ≥ 80 cm for European males and females respectively, and any two of the following four components: systolic blood pressure (BP) ≥ 130 mmHg or diastolic BP ≥ 85 mmHg or treatment of hypertension, triglycerides (TG) ≥ 1.7 mmol/l, HDL-cholesterol (HDL-C) < 1.29 mmol/l in females, and < 1.03 mmol/l in males or treatment for lipid abnormality, fasting glucose ≥5.6 mmol/l or previously diagnosed T2DM. WC and Waist-to-Height-Ratio (WHtR) > 0.5 are proxy measures of abdominal obesity [28, 29].

In addition, an echocardiography (GE Medical Systems, Vivid S6), as well as a standard 12-leads ECG at rest (EINTHOVEN, GOLDBERGER, WILSON leads) was performed and recorded on CUSTO CARD (custo med GmbH, Munich) as well as a Holter ECG (CUSTO FLASH 500 Multiday, custo med GmbH, Munich) for at least 18 h. ECG at rest and Holter ECG were analysed computer-assisted using Minnesota ECG Code Classification [30]. The SVPBs and VPBs were detected by software and confirmed by 2 cardiologists.

Echocardiographic studies were performed by 2 cardiologists in standard views [31]: parasternal long-axis view of the left ventricle in 2D, colour Doppler and M-mode, parasternal RV inflow- and outflow tract view in 2D and coulour Doppler, apical four- and five chamber view in 2D and colour Doppler, apical two-chamber view in 2D and colour Doppler, apical long-axis view in 2D and colour doppler, transmitral, transaortic and tricuspid velocities as well as tissue Doppler on mitral annulus (lateral velocities) in 4-chamber-view in accordence to the recommendations [32]. The upper cut off for LVMI in men is ≥115 g/m^2^ and women ≥95 g/m^2^ [7]. All echo parameters including LVMI were created by Vivid 6, GE Medical Systems, Ver. 11.2.0 b. 40.

**Fig. 2:**
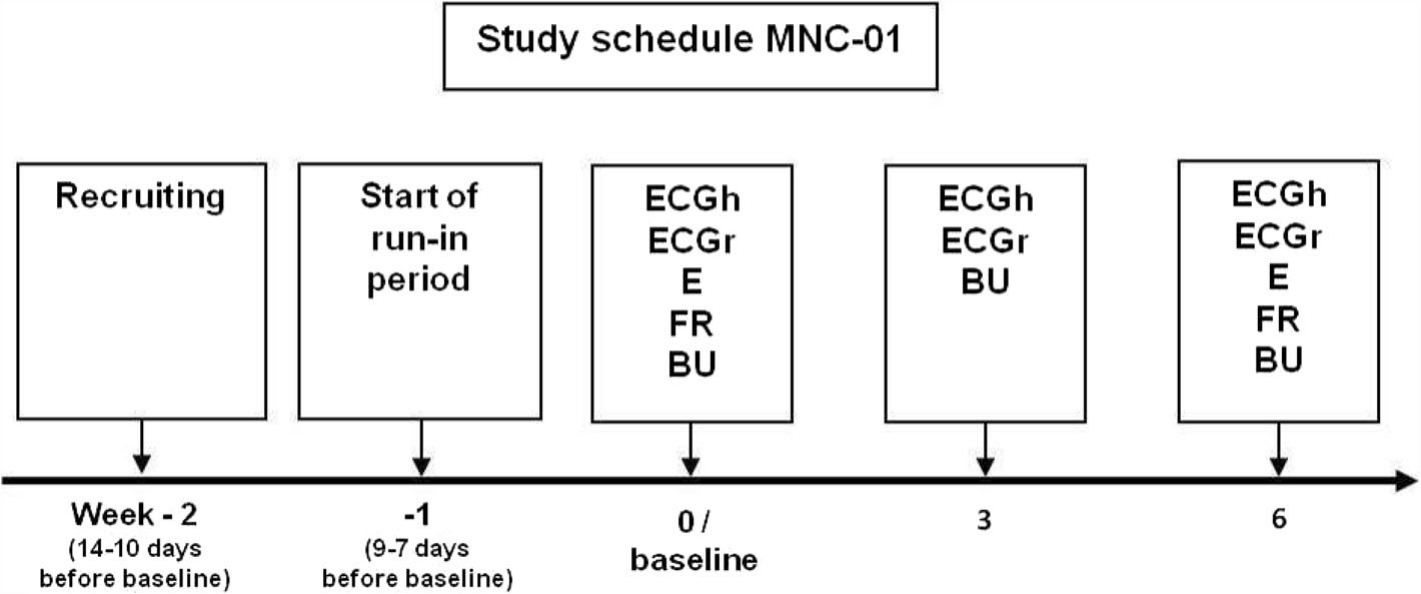
Study schedule. Recruiting / Start of run-in period: At the first visit the patients were informed about the study in detail and the necessary examinations for inclusion and exclusion criteria were carried out. If the criteria were met and the patient gave his written consent to participate in the study, the placebo tablets for the run-in phase were issued at the second visit. Baseline: Inclusion and exclusion criteria were once again checked, then the patients were randomized. In addition anthropological parameters were collected at each visit. Abbreviations used: E, echocardiography; ECG, electrocardiogramm; ECGr, ECG at rest; ECGh, Holter ECG; FR, 3-day food record; BU, Blood and urinary samples

Blood and urinary samples for biochemical assessment were collected at baseline, after 3 and after 6 weeks of dietary intervention (Fig. 2).

### Laboratory tests

All laboratory analyses were conducted by Laboratory Schottdorf MVZ GmbH, Augsburg, Germany. The Analyses of serum glucose (hexokinase method), gammaglutamyltransferase (IFCC method), glycated haemoglobin (HbA1c) (turbidimetric immunologic inhibition assay (TINIA)), insulin (ECLIA), total cholesterol (TC) (CHOD-PAP method), LDL-C and HDL-C (enzymatic colour test), TG (GPO-PAP method) and CRP sensitive (turbidimetry) were performed on a Roche analyser. 24 h urinary magnesium and potassium excretion was analysed by atomic absorption spectrometry. The HOMA is a measure for IR. HOMA-IR was calculated from insulin and glucose concentrations.

### Statistical analysis

Statistical comparisons were made between groups using the nonparametric Mann-Whitney-U-test. The nonparametric Wilcoxon test was used for data comparison at different time points within groups. Differences in classified variables were tested by Fisher’s exact test. The influence of covariates was analysed by analysis of variance (ANOVA). All statistical tests were based on per protocol population and two-sided. Differences were considered significant at *p* < 0.05. Data are reported as mean ± standard deviation (SD). Linear regression modelling was performed to detect the changes of different components of the cardiometabolic parameters as a function of LVMI and serum magnesium concentration. The effect size Cohen’s d is defined as the difference between two means divided by a SD for the data. Effect size (ES) threshold: small 0.20, medium 0.50, large 0.80, very large 1.30 [33]. The first and third quartile (Q1 and Q3 respectively) are shown. All analyses were conducted using SPSS® for Windows (version 22.0).

## Results

### Patients’ characteristics

Fifty-four patients were evaluated per protocol, 29 in verum group, and 25 in placebo group (Fig. 1). Six patients did not finish the study per protocol: One patient in the placebo group has had an artificial hyperkalemia. 5 patients of the study population consumed less than 95% of study administration (8%), 4 of these patients were in placebo group.

No serious side effects were reported under dietary intervention. One patient in the verum group and one in the placebo group reported gastric pain. This could be caused by supplement intake. The supplementation has not been interrupted.

### Pre-existing diseases und medications

Arterial hypertension (AH) was the most frequent pre-existing disease (65.5% verum group, 52.0% placebo group). Coronary artery disease (CAD) was present in 27.6% of patients in the verum group, and in 28.0% in the placebo group. A long-term medical treatment with beta blocking agents, ACE-inhibitors/AT II receptor antagonists, calcium channel blocker as well as spironolactone was given to 21 patients (72.4%) in the verum group and 16 patients (64.0%) in the placebo group. T2DM was found in 10.3% of patients in the verum group and 8.0% in the placebo group. At baseline 68.5% of patients had a dyslipidemia (verum 68.9%, placebo 68.0%). Treatment with statins was recorded in 34.5% in the verum group and 28.0% in the placebo group. Further baseline characteristics of both groups are shown in Table 1.

**Table 1:** Baseline characteristics

	Verum (*n* = 29)	Placebo (*n* = 25)	V vs. P
	x ± SD	x ± SD	*p*-value
Sex (n/%)			
female	14 (48.3%)	13 (52.0%)	1.000
male	15 (51.7%)	12 (48.0%)	
Age (years)	59.7 ± 10.2	59.8 ± 13.8	0.524
Height (cm)	171.9 ± 8.8	171.0 ± 9.1	0.740
Weight (kg)	82.0 ± 17.9	82.9 ± 14.7	0.561
BMI (kg/m^2^)	27.6 ± 4.8	28.3 ± 4.2	0.535
< 25.0 kg/m^2^ (n/%)	10 (34.5%)	7 (28.0%)	0.940
25.0–29.9 kg/m^2^ (n/%)	11 (37.9%)	11 (44.0%)	
≥ 30.0 kg/m^2^ (n/%)	8 (27.6%)	7 (28.0%)	
WC female (cm)	88.9 ± 13.1	87.6 ± 9.6	0.847
≥ 88 cm (n/%)	9 (31.0%)	5 (20.0%)	0.449
WC male (cm)	103.6 ± 14.6	106.6 ± 8.8	0.286
≥ 102 cm (n/%)	8 (27.6%)	9 (36.0%)	0.429
WHtR	0.558 ± 0.080	0.565 ± 0.064	0.602
≥ 0,5 (n/%)	21 (72.4%)	20 (80.0%)	0.545
BP systolic (mmHg)	142.2 ± 17.4	134.7 ± 12.5	0.077
≥ 130 mmHg (n/%)	23 (79.3%)	16 (64.0%)	0.239
BP diastolic (mmHg)	86.3 ± 9.7	82.2 ± 8.6	0.160
≥ 85 mmHg (n/%)	16 (55.2%)	9 (36.0%)	0.182
Heart rate/min	71.4 ± 12.5	72.9 ± 12.7	0.773

*Abbreviations: BMI* body mass index, *WC* waist circumference, *WHtR* waist to height ratio, *BP* blood pressure

### Metabolic syndrome and its components

Body mass index (BMI) ≥ 25 kg/m^2^ was recorded in 65.5% of the verum group and in 72.0% of the placebo group at baseline. An abdominal obesity with WHtR ≥0.5 was found in 72.4% of the verum group and in 80.0% of the placebo group (Table 1). The number of patients with criteria of MetS according to IDF compared to baseline to the study end decreased in the verum group of 16 to 15, and on the contrary in the placebo group increased from 8 to 12. At baseline 5 patients (17.2%) in the verum group and 4 (16.0%) in the placebo group had an atherogenic dyslipidemia and at study end 4 patients in the verum group (13.8%) and 3 (12.0%) in the placebo group.

### LV function

In all patients at baseline the right ventricular end diastolic diameter (RVEDD), left atrium (LA), left ventricular end systolic diameter (LVESD), left ventricular end diastolic diameter (LVEDD), left ventricular ejection fraction (LVEF), intraventricular septum (IVS) and left ventricular posterior wall (LVPW) were in normal range. However, patients with LVEF 54–65% showed a significant increase of LVEF (*p* = 0.020, ES = 0.995, Q1;Q3: 0.5;15.0) in the verum group after intervention (Table 2).

**Table 2:** Changing of patient’s characteristics: comparison of echocardiographic values before and after dietary intervention

	Verum (*n* = 29) x ± SD			Placebo (*n* = 25) x ± SD		
	Baseline	Week 6	Diff	Baseline	Week 6	Diff
LA (mm)	35.8 ± 5.9	37.1 ± 5.0	1.39 ± 2.92^#^	36.4 ± 6.4	35.8 ± 6.1	− 0.68 ± 4.26
RVEDD (mm)	26.8 ± 5.3	26.3 ± 3.4	− 0.52 ± 4.67	25.2 ± 4.1	25.1 ± 3.5	− 0.08 ± 4.29
LVEDD (mm)	49.0 ± 5.7	47.6 ± 7.1	− 1.34 ± 5.47^#^	47.9 ± 6.9	48.4 ± 7.5	0.52 ± 5.64
LVESD (mm)	30.4 ± 4.9	30.1 ± 5.5	− 0.31 ± 3.27	29.5 ± 5.2	30.2 ± 6.7	0.72 ± 4.20
LVEF (%)	68.6 ± 8.3	70.2 ± 9.0	1.66 ± 8.94	70.0 ± 9.3	70.3 ± 8.9	0.28 ± 10.81
< 65% (n/%)	10 (34.5%)	9 (31.0%)	–	6 (24.0%)	7 (28.0%)	–
< 65%	59.5 ± 4.2	66.8 ± 9.5	7.30 ± 8.22^#^	57.5 ± 5.4	64.5 ± 11.8	7.00 ± 7.75
≥ 65%	73.5 ± 5.5	72.5 ± 8.4	− 1.00 ± 8.07	74.0 ± 6.3	72.1 ± 7.3	− 1.84 ± 10.93
IVS (mm)	10.6 ± 1.6	9.9 ± 1.9	− 0.62 ± 1.78	10.6 ± 1.4	10.2 ± 1.5	− 0.44 ± 1.32
LVPW (mm)	10.7 ± 1.6	10.3 ± 1.6	− 0.41 ± 1.52	10.4 ± 1.5	10.2 ± 1.6	− 0.20 ± 1.12
E/E´ > 8 (n/%)	2 (25.0%)	3 (37.5%)	–	2 (33.3%)	0 (0.0%)	–
E wave	0.73 ± 0.19	0.71 ± 0.16	− 0.02 ± 0.18	0.73 ± 0.18	0.72 ± 0.15	− 0.01 ± 0.15
A wave	0.74 ± 0.24	0.68 ± 0.28	− 0.06 ± 0.19	0.71 ± 0.18	0.73 ± 0.15	0.01 ± 0.16
E/A ratio	1.05 ± 0.35	1.18 ± 0.49	0.13 ± 0.38^#^	1.45 ± 2.04	1.02 ± 0.21	− 0.42 ± 2.04
LVMI g/m2	98.2 ± 24.4	90.0 ± 37.0	−8.15 ± 35.48^##^	93.5 ± 26.5	90.9 ± 25.9	−2.61 ± 21.20
LVMI g/m^2^ female^a^	87.2 ± 16.6	77.7 ± 20.9	−9.5 ± 15.6#	89.0 ± 26.2	80.2 ± 21.8	−8.7 ± 23.1
LVMI g/m^2^ male^b^	108.4 ± 26.4	101.5 ± 45.1	−6.9 ± 47#	98.4 ± 27.1	102.4 ± 25.9	4.0 ± 17.5

Abbreviations: LA, left atrium; RVEDD, right ventricular end diastolic diameter; LVEDD, left ventricular end diastolic diameter; LVESD, left ventricular end systolic diameter; LVEF, left ventricular ejection fraction; IVS, interventricular septum; LVPW, left ventricular posterior wall; E/A ratio, ratio of the early (E) to late (A) ventricular filling velocities; LVMI, Left ventricular mass index

^a^*n* = 14 (verum)/*n* = 13 (placebo, ^b^ *n* = 15 (verum)/*n* = 12 (placebo)

# *p* < 0.05 within the group, ## *p* < 0.01 within the group

After dietary intervention the LV mass index (LVMI) was significantly reduced in the verum group (*p* = 0.003, ES = 0.260, Q1;Q3: -26.5;-2.2). If a distinction is made between male and female this parameter is significantly reduced too, especially by reducing of this parameter in woman (*p* = 0.035). The LVEDD (*p* = 0.050, ES = 0.210, Q1/Q3: -5.0; 1.0) were reduced and E/A ratio (*p* = 0.051, ES = 0.401, Q1; Q3: -0.04; 0.27) in normal range was improved. In the placebo group these results were not found. But the differences between these both groups are statistical not significant.

The linear regression analysis showed that LVMI in the verum group depended on the initial value and the magnesium concentration in the serum during the course of the study. The change of LVMI is depending on the alteration in HDL-C and diastolic blood pressure. Only in the verum group the favourable changes of LVMI were found under this condition. In contrast, these results could not be shown in the placebo group (Table 3).

**Table 3:** Changing of LVMI depending on alteration in HDL-Cholesterol and diastolic blood pressure

	total p_ANOVA_	Depending variable: Regression coefficient B (*p*-value)			
		LVMI Baseline	Serum-Mg Week 6	Changing of	
Verum (*n* = 29)	0,008	0,014	0,002	HDL-C	−0,567^1^ (0,507)
	0,003	0,011	0,004	BP diastolic	1,623^2^ (0,129)
Placebo (*n* = 25)	0,069	0,014	0,257	HDL-C	0,697 (0,283)
	0,109	0,020	0,421	BP diastolic	−0,164 (0,780)

Modell of linear regression, depending variable: changing of LVMI, age adjusted

*Abbreviations: LVMI* left ventricular mass index, *Serum-Mg* serum magnesium, *HDL-C* HDL cholesterol, *BP* blood pressure

^1^Increase of HDL-C correlates with decrease of LVMI

^2^Decrease of BP diastolic correlates with decrease of LVMI

The interventricular septal thickness was reduced in tendency only in the verum group (*p* = 0.053, ES = 0.348, Q1; Q3: -2.0;0.5), while LVPW was unchanged in both groups. On the other hand, the LA parameters were significantly increased in the verum group after intervention but within the normal range (*p* = 0.018, ES = 0.314, Q1; Q3: -0.75; 3.75) (Table 2). Furthermore could be demonstrated a strong correlation between LVMI and LA in men (*p* = 0.018), but not in female (*p* = 0.225).

When looking at the results broken down by HOMA quartiles and parameters of LV function it can be noted, that in female diameter of LA increases age-adjusted significantly with the quartiles (*p* = 0.001) and in male the LVEDD (*p* = 0.055) (Table 4). The influence of HOMA-quartiles was proofed by a regression model.

**Table 4:** HOMA-quartiles for LVMI, LA, IVS, PW, and LVEDD in male and female

HOMA-quartile	1: 0.7 … 1.5	2: 1.6 … 2.4	3: 2.5 … 4.3	4: > 4.3	*p*-value
N (male/female)	7/7	5/8	5/9	10/3	
LVMI (male)	96.1 +/− 36.8	106.2 +/− 23.2	102.9 +/− 35.9	108.9 +/− 16.4	0.372
LVMI (female)	80.6 +/− 19.4	95.7 +/− 25.1	90.7 +/− 21.4	78.6 +/− 13.5	0.967
LA (male)	37.3 +/− 7.6	38.0 +/− 7.2	37.0 +/− 4.8	41.4 +/− 4.2	0.118
LA (female)	29.1 +/− 2.5	32.0 +/− 2.6	34.0 +/− 5.6	40.3 +/− 4.0	0.001
IVS (male)	11.6 +/− 1.8	11.2 +/− 1.3	10.8 +/− 1.8	11.0 +/− 1.3	0.533
IVS (female)	9.6 +/− 1.4	9.8 +/− 1.3	10.8 +/− 1.2	9.3 +/− 1.2	0.489
PW (male)	11.3 +/− 1.9	11.4 +/− 1.5	11.0 +/− 2.0	11.3 +/− 1.4	0.880
PW (female)	9.4 +/− 1.5	9.6 +/− 0.7	10.3 +/− 1.4	10.0 +/− 1.0	0.205
LVEDD (male)	47.0 +/− 8.5	50.0 +/− 7.1	51.4 +/− 6.7	53.5 +/− 4.1	0.055
LVEDD (female)	43.1 +/− 4.8	49.8 +/− 4.5	44.9 +/− 4.5	47.3 +/− 3.2	0.562

*Abbreviations: HOMA* Homeostasis Model Assessment, *LVMI* left ventricular mass index, *LA* left atrium, *IVS* interventricular septum, *PW* posterior wall, *LVEDD* left ventricular end diastolic diameter

### HOMA-IR

In the verum group abnormal fasting plasma glucose ≥5.6 mmol/l was distinctly reduced after 6 weeks of dietary intervention compared to baseline (*p* = 0.055, ES = 0.388, Q1;Q3: -1.30;-0.11), in the placebo group this difference was not found (*p* = 0.328, ES = 0.604, Q1;Q3: -0.94;0.28).

At baseline, elevated fasting insulin concentration (≥ 58 pmol/l) was recorded in 19 patients of the verum group and in 17 patients of the placebo group, a normal level (< 58 pmol/l) in 10 patients in the verum group vs. 8 patients in the placebo group. But only in the verum group it was in tendency reduced after intervention (*p* = 0.053, ES = 0.232, Q1;Q3: -35.1;9.4), and significantly in patients with fasting insulin ≥58 pmol/l (*p* = 0.020, ES = 0.340, Q1;Q3: -42.7; 1.4).

Elevated HOMA-IR > 2.5 was found in 14 patients in the verum group, and 11 patients in the placebo group. An euglycaemic IR (FPG < 5.6 mmol/l, HOMA-IR ≥ 2.5) showed at baseline 8 patients in the verum group and 4 patients in the placebo group. HOMA-IR was in tendency reduced in the verum group (*p* = 0.053, ES = 0.218, Q1;Q3: -1.25;0.25) at the end of study, particularly for patients with HOMA-IR > 2.5 in the verum group (*p* = 0.068, ES = 0.340, Q1;Q3: -2.90;-0.22) (Table 5). At the study end only 3 patients had a HOMA-IR ≥ 2.5 (*p* = 0.063), and in the placebo group 2 patients a HOMA-IR ≥ 2.5 (*p* = 0.625).

**Table 5:** Changing of patient’s characteristics: comparison of biochemical values before and after dietary intervention

	Verum (*n* = 29) x ± SD			Placebo (*n* = 25) x ± SD		
	Baseline	Week 6	Diff	Baseline	Week 6	Diff
CrP (nmol/l)	26.0 ± 28.7	24.8 ± 22.6	−1.22 ± 23.37	20.8 ± 19.8	29.8 ± 42.1	9.07 ± 39.52
< 10 nmol/l (n/%)	9 (31.0%)	9 (31.0%)	–	10 (40.0%)	12 (48.0%)	–
10–30 nmol/l (n/%)	13 (44.8%)	13 (44.8%)		10 (40.0%)	6 (24.0%)	
> 30 nmol/l (n/%)	7 (24.1%)	7 (24.1%)		5 (20.0%)	7 (28.0%)	
FPG (mmol/l)	5.49 ± 1.58	5.30 ± 1.28	− 0.20 ± 0.75	5.35 ± 0.65	5.15 ± 0.77	−0.20 ± 0.63^#^
< 5.6 mmol/l	4.76 ± 0.54	4.79 ± 0.62	0.03 ± 0.39	5.04 ± 0.27	4.91 ± 0.70	− 0.13 ± 0.58
≥ 5.6 mmol/l	7.12 ± 1.94	6.42 ± 1.67	−0.70 ± 1.10	6.15 ± 0.65	5.79 ± 0.56	−0.36 ± 0.75
HbA1c (%)	5.77 ± 0.68	5.76 ± 0.63	−0.01 ± 0.35	5.64 ± 0.39	5.66 ± 0.33	0.02 ± 0.39
F Insulin (pmol/l)^a^	113.9 ± 127.5	89.5 ± 75.8	− 24.4 ± 73.4	83.6 ± 44.9	71.3 ± 51.3	−12.3 ± 54.0
< 58 pmol/l (n/%)	10 (34.5%)	11 (37.9%)	–	8 (32.0%)	10 (40.0%)	–
< 58 pmol/l	39.2 ± 9.9	44.7 ± 18.4	5.2 ± 19.5	41.3 ± 13.5	40.4 ± 13.3	− 0.9 ± 16.0
≥ 58 pmol/l	153.1 ± 143.2	113.1 ± 84.0	−39.9 ± 86.3^#^	103.5 ± 40.0	85.9 ± 56.3	− 17.7 ± 64.6
HOMA-IR^a^	4.93 ± 9.18	3.39 ± 4.00	− 1.54 ± 5.69^#^	2.97 ± 1.86	2.50 ± 2.17	−0.47 ± 2.26
≤ 2.5 (n/%)	15 (51.7%)	20 (69.0%)	–	14 (56.0%)	18 (72.0%)	–
≤ 2.5	1.55 ± 0.52	1.57 ± 0.59	0.03 ± 0.56	1.66 ± 0.52	1.95 ± 2.19	0.29 ± 2.02
> 2.5	8.56 ± 12.40	5.34 ± 5.12	− 3.22 ± 7.97	4.64 ± 1.60	3.20 ± 2.03	−1.44 ± 2.28
Na (mmol/l)	143.0 ± 4.5	142.2 ± 2.7	−0.76 ± 4.25	141.9 ± 2.5	141.6 ± 2.8	−0.28 ± 2.51
< 140 mmol/l	138.0 ± 1.2	139.0 ± 3.5	1.00 ± 2.83	138.4 ± 0.9	138.8 ± 4.4	0.40 ± 4.22
≥ 140 mmol/l	143.8 ± 4.3	142.8 ± 2.2	− 1.04 ± 4.41	142.8 ± 2.0	142.4 ± 1.7	− 0.45 ± 2.01
K (mmol/l)	4.44 ± 0.53	4.55 ± 0.43	0.11 ± 0.44	4.59 ± 0.37	4.50 ± 0.31	−0.09 ± 0.33*
< 4.0 mmol/l	3.72 ± 0.17	4.08 ± 0.34	0.35 ± 0.26	3.70	4.10	0.40
≥ 4.0 mmol/l	4.56 ± 0.47	4.62 ± 0.40	0.07 ± 0.45	4.63 ± 0.33	4.52 ± 0.31	− 0.11 ± 0.32
Mg (mmol/l)	0.845 ± 0.069	0.858 ± 0.068	0.013 ± 0.047	0.874 ± 0.084	0.851 ± 0.068	−0.024 ± 0.056^#^**
< 0.75 mmol/l	0.727 ± 0.023	0.773 ± 0.021	0.048 ± 0.035	0.72	0.77	0.05
≥ 0.75 mmol/l	0.859 ± 0.058	0.867 ± 0.068	0.009 ± 0.047	0.881 ± 0.079	0.854 ± 0.068	− 0.027 ± 0.055^##^**
Cl (mmol/l)	102.0 ± 3.4	100.9 ± 2.6	−1.03 ± 3.80	101.4 ± 2.6	101.4 ± 3.2	0.00 ± 3.20
< 100 mmol/l	98.0 ± 1.2	99.6 ± 2.4	1.57 ± 2.51	97.8 ± 1.0	100.2 ± 2.5	2.33 ± 2.88
≥ 100 mmol/l	103.2 ± 2.8	101.4 ± 2.5	−1.86 ± 3.81^#^	102.6 ± 1.8	101.8 ± 3.4	−0.74 ± 3.00
Creatinine (μmol/l)	77.4 ± 14.6	76.4 ± 14.9	−0.95 ± 6.98	76.6 ± 17.4	75.3 ± 16.4	−1.31 ± 7.02
GFR (ml/min)	83.6 ± 13.9	85.4 ± 14.9	1.79 ± 8.09	86.1 ± 16.0	86.8 ± 16.3	0.76 ± 6.48
TC (mmo/l)	4.97 ± 1.00	5.12 ± 1.09	0.15 ± 0.66	5.25 ± 0.94	5.10 ± 1.04	−0.15 ± 0.69
≥ 5.2 mmol/l	5.74 ± 0.63	6.08 ± 0.79	0.34 ± 0.85	5.86 ± 0.75	5.89 ± 0.67	0.03 ± 0.37
LDL-C (mmol/l)	3.14 ± 0.97	3.10 ± 0.83	− 0.04 ± 0.44	3.39 ± 0.92	3.30 ± 0.97	−0.09 ± 0.45
< 2.6 mmol/l	2.02 ± 0.27	2.27 ± 0.48	0.25 ± 0.45	2.11 ± 0.56	2.33 ± 0.32	0.22 ± 0.25
≥ 2.6 mmol/l	3.59 ± 0.76	3.43 ± 0.70	−0.15 ± 0.40	3.64 ± 0.76	3.48 ± 0.94	−0.15 ± 0.46
< 3.3 mmol/l	2.43 ± 0.48	2.59 ± 0.51	0.16 ± 0.36	2.72 ± 0.57*	2.67 ± 0.40	− 0.05 ± 0.33
≥ 3.3 mmol/l	4.08 ± 0.55	3.78 ± 0.68	− 0.30 ± 0.41^#^	4.01 ± 0.71	3.88 ± 0.99	− 0.14 ± 0.54
HDL-C (mmol/l)	1.40 ± 0.41	1.39 ± 0.43	−0.01 ± 0.17	1.45 ± 0.47	1.40 ± 0.42	−0.05 ± 0.17
TG (mmol/l)	1.56 ± 0.77	1.97 ± 1.86	0.42 ± 1.30^#^	1.34 ± 0.53	1.36 ± 0.50	0.02 ± 0.58
≥ 1.7 mmol/l	2.63 ± 0.57	3.71 ± 2.95	1.08 ± 2.41	2.08 ± 0.28*	1.71 ± 0.61	−0.37 ± 0.76
Uric acid (μmol/l)	338 ± 90	331 ± 80	− 7.2 ± 42.1	315 ± 100	308 ± 91	− 6.9 ± 39.9
Gamma-GT (μkat/l)	0.64 ± 0.83	0.63 ± 0.72	−0.01 ± 0.17	0.53 ± 0.29	0.52 ± 0.26	−0.01 ± 0.17
ASAT (μkat/l)	0.42 ± 0.20	0.42 ± 0.11	0.00 ± 0.14	0.40 ± 0.11	0.40 ± 0.13	0.00 ± 0.09
ALAT (μkat/l)	0.47 ± 0.24	0.46 ± 0.21	− 0.02 ± 0.16	0.43 ± 0.26	0.45 ± 0.28	0.02 ± 0.11
TSH (μIU/ml)	2.15 ± 1.17	1.96 ± 1.06	−0.19 ± 0.52^#^	2.19 ± 1.20	1.92 ± 1.00	−0.28 ± 0.57^#^
24 h urinary collection						
Na (mmol/24 h)	193.7 ± 73.6	186.7 ± 76.0	−7.0 ± 79.7	167.9 ± 53.5	177.4 ± 64.1	9.5 ± 46.8
K (mmol/24 h)	68.2 ± 25.3	76.6 ± 26.2	8.4 ± 28.0	67.3 ± 19.3	71.6 ± 22.5	4.2 ± 18.0
K i.S./K (24 h)	0.072 ± 0.023	0.067 ± 0.027	− 0.005 ± 0.028	0.076 ± 0.034	0.069 ± 0.021	−0.008 ± 0.030
Mg (mmol/24 h)	4.03 ± 1.14	4.79 ± 1.79	0.76 ± 1.85^#^	3.78 ± 1.45	3.86 ± 1.88	0.08 ± 1.75*
Mg i.S./Mg (24 h)	0.227 ± 0.070	0.221 ± 0.162	−0.006 ± 0.180	0.260 ± 0.095	0.268 ± 0.121	0.008 ± 0.093
Cl (mmol/24 h)	175.6 ± 70.2	174.9 ± 76.9	−0.72 ± 74.63	153.5 ± 54.9	161.2 ± 70.7	7.72 ± 43.40
Albumin (mg/24 h)	10.8 ± 19.2	12.0 ± 26.4	1.17 ± 15.42	9.5 ± 17.2	11.7 ± 18.8	2.24 ± 5.88
Albumin/crea (24 h)	0.82 ± 1.40	0.80 ± 1.36	−0.02 ± 0.36	0.77 ± 1.31	0.96 ± 1.55	0.20 ± 0.48

*Abbreviations: HK* haematocrit, *MCHC* mean corpuscular/cellular haemoglobin concentration, *MCV* mean corpuscular/cell volume, *CrP* C reactive protein, *FPG* fasting plasma glucose, *HbA1c* glycated haemoglobin A1c, *F Insulin* fasting insulin, *HOMA-IR* homeostasis model assessment-insulin resistance, *Na* natrium, *K* potassium, *MG* magnesium, *Cl* chloride, *GFR* glomerular filtration rate, *TC* total cholesterol, *LDL-C* LDL-cholesterol, *HDL-C* HDL-cholesterol, *TG* triglycerides, *ASAT* aspartate-aminotranferase, *ALAT* alanin-aminotranferase, *TSH* thyroid stimulating hormone, # *p* < 0.05 within the group, ## *p* < 0.01 within the group, * *p* < 0.05 between the groups, ** *p* < 0.01 between the groups, ^a^Large variation coefficients indicate statistical outliers. By sensitivity analyses the influence of these statistical outliers was checked. The results in the full analysis set were confirmed

### Other biochemistry cardiovascular risk factors

All relevant biochemical parameters are summarized in Table 5. In the blood cell count up to a significant reduction of leucocytes in normal range in the verum group (*p* < 0.05) all components were in normal range in the verum as well as the placebo group. Total cholesterol (TC) was unchanged in verum and placebo group after dietary intervention. LDL-cholesterol (LDL-C) was nearly unchanged in the verum group, and in the placebo group slightly reduced; HDL-C was unchanged in both groups. After dietary intervention TG were significantly increased in the verum group (*p* = 0.011, ES = 0.292, Q1;Q3: -0.01;0.30), but unchanged in the placebo group. TG concentrations ≥1.7 mmol/l were found at baseline in 27.6% of patients in the verum group and 28.0% in the placebo group. At study end this relation was 31.0 to 20.0%.

Renal function measured by creatinine and glomerular filtration rate (GFR) was normal in all patients. The changes in serum magnesium was significantly higher compared to the placebo group after intervention (*p* = 0.005, ES = 0.708, Q1;Q3: -0.02;0.05 vs. -0.06;0.01). The serum potassium level was significantly increased after intervention in the verum group (*p* = 0.034, ES = 0.512, Q1;Q3: 0.0;0.3 vs. -0.5;0.1), compared to the placebo group. However, the increase of both serum magnesium as well as potassium after intervention was in normal range.

In 24 h urinary collection potassium excretion (*p* = 0.059, ES = 0.326, Q1;Q3: -7.2;30.2) as well as magnesium excretion were elevated (*p* = 0.018, ES = 0.507, Q1;Q3: -0.18;1.66) in the verum group. The magnesium excretion was significant compared to the placebo group too (*p* = 0.036, ES = 0.377, Q1;Q3: -0.13;1.51 vs. -0.64;0.46). This result was an effect of compliance. The normal renal function could be shown in 24 h urinary collection: albumin excretion as well as the quotient albumin/creatinine/24 h was in normal range. Only in the verum group this quotient was in tendency reduced after dietary intervention, while in the placebo group this quotient was increased. This difference in both groups was reduced (*p* = 0.083, ES = 0.521, Q1;Q3: -0.09;0.03 vs. -0.05;0.22) .

### Rhythm disturbances

In this pilot study a reduction of PBs could be observed in the verum group, but this result was not significant, probably due to the small number of patients and an enormous variance of the PBs. On the other hand, in the placebo group the reduction of VPBs was remarkable with the same enormous variance as in the verum group (*p* = 0.005, ES = 0.369, Q1;Q3: -935.0;5.0). The heart rate was decreased only in the verum group (*p* = 0.027, ES = 0.256, Q1;Q3: -7.8;0.8) (Table 6).

**Table 6:** Changing of patient’s characteristics: comparison of Holter ECG before and after dietary intervention

	Verum (*n* = 29) x ± SD			Placebo (*n* = 25) x ± SD		
	Baseline	Week 6	Diff	Baseline	Week 6	Diff
VPBs > 500/24 h	2486 ± 2133	2316 ± 3232	− 170 ± 2469	5717 ± 7662	2649 ± 3287	− 3068 ± 5259^##^
SV tachycardia (hr > 100/min)	37.3 ± 66.1	23.7 ± 46.0	− 13.6 ± 67.4	52.2 ± 67.3	39.9 ± 103.8	− 12.4 ± 96.9
SVPBs > 200/24 h	2000 ± 1874	2204 ± 1474	204 ± 1541	1025 ± 406	585 ± 786	− 440 ± 427
SVPBs < 200/24 h with symptoms	20.5 ± 32.9	21.3 ± 46.9	0.85 ± 55.83	39.2 ± 36.4*	107.7 ± 298.0	68.6 ± 269.7
Heart rate (bpm)	71.4 ± 12.5	68.4 ± 10.8	− 3.0 ± 7.9#	72.9 ± 12.7	70.3 ± 13.2	−2.6 ± 9.3

*Abbreviations: VPBs* ventricular premature beats, *SV* supraventricular, *SVPBs* supraventricular premature beats

#*p* < 0.05 within the group, ##*p* < 0.01 within the group, *p < 0.05 between the groups

A change of awareness of symptoms measured by a visual analogue scale (VAS points) also could not be found.

## Discussion

### Metabolic syndrome and its components

The MetS is the burden of our century and in Germany could be found with prevalence depending on the region between 14 and 23% [34].

Precursors of MetS like disturbed glucose tolerance, triggered by elevated fasting insulin followed by IR, lead already at early stage to structural changes of the left ventricle and influence on diastolic function. These factors seem to be acting independently from CAD associated especially with diastolic dysfunction or heart failure [4, 5, 35]. Our knowledge based on experimental [36] and clinical studies concerning the MetS [4, 5, 37, 38] as well as meta-analyses [28].

The influence of micronutrients on these pathological processes in MetS is only partially known. There is evidence that magnesium plays an important role, especially in the glucose metabolism [10]. In addition, a positive effect on glucose metabolism is discussed for magnesium and for coenzyme Q10 [21, 24]. The effects of coenzyme Q10 on the glucose metabolism may be based on multifactorial mechanisms i.e. by reduction of oxidative stress [39, 40].

On average, the patients enrolled in this study were overweight, with abdominal obesity measured by WC and also by WHtR. These basic components of MetS as well as fasting plasma glucose were unchanged after intervention. The reduction of systolic blood pressure in the verum group at study end has not influenced this result. Therefore we did not found a reduction of the components of MetS in verum group. The results of this post hoc analysis seem to suggest that this specific combination of micronutrients improves the cardiometabolic profile in patients with CA.

### LV function

The MetS and its components – IR/T2DM, AH and abdominal obesity – are associated with impaired mitochondrial function and increased oxidative stress. These pathophysiological mechanisms contribute to the development of diastolic dysfunction [4, 35, 37, 38]. Ikee et al. [41] found that diastolic dysfunction at first is an asymptomatic disorder of relaxation and/or compliance associated with normal systolic pump function. It depends on preload, end diastolic pressure and structure of the LV wall.

We found signs of preclinical LV diastolic dysfunction parallel to metabolic changes. This is consistent with the results of this pilot study in comparison of the verum and placebo group. At baseline the E/A ratio was in tendency lower in the verum group than in the placebo group. After intervention E/A ratio was significantly improved in the verum group, but not in the placebo group. Diastolic dysfunction assessed with the use of E/A worsened with the number of components of MetS [42]. The changes in diastolic function are age related [32, 42, 43] and furthermore depending on components of MetS like overweight, T2DM, AH, and CAD [4, 35]. Furthermore, the present study showed that the LVEF could only be improved if it was within the lower normal range (54–65%). Magnesium leads to an economization of cardiac pump function [44]. In a review it could be shown that also CoQ10 improves parameters of heart function like ejection fraction in heart failure [45]. This could be confirmed with this study: the results demonstrate a significant improvement of EF ≤ 65% in the verum group, but this effect was not significant between the both study groups (Table 2).

Different authors described that the prevalence of LV diastolic dysfunction measured by diameters of the LA, left ventricle, and LVMI is significantly increased the more components of MetS exist [4, 5, 7].

In the verum group of the present study the diameter of LA was increased and especially related to the HOMA quartiles, but no more than in upper limit of normal. In male but not in female a strong positive correlation between LVMI and LA at baseline as a sign of diastolic dysfunction was demonstrable. In contrary in the placebo group this diameter was reduced. To what extent the SVPBs are relevant in this process is yet unknown. Measured by effect size functional parameters are more improved in the verum group within 6 weeks than structural parameters like LVMI. Overall in literature the findings in disturbances in glucose metabolism and transthoracic echocardiographic evaluated structural and functional parameters are not consistent. This may be based on exclusion of older and patients with higher BMI [46]. In accordance with Rutter et al. [46] and Hwang et al. [47] we found these relations to different parameters of LV diastolic dysfunction by transthoracic echocardiography. Similar results on LV function in patients with insulin resistance and glycaemic abnormalities were described by Velagaleti et al. [48] by means of MR imaging. These associations were more frequently significant in males. Overall is the reduction of LVMI due to intervention within 6 weeks an interesting finding. The mechanisms of modification of LVMI are not yet fully understood. It is accepted that arterial hypertension is contributing to the development of LV hypertrophy. But the changes in 24-h blood pressure measuring contribute only 25 to 30% of variation of LV mass [49]. Sundström et al. [50] described in 2000 that different parameters of MetS stronger related to the LV wall thickness and concentric remodelling than to LV hypertrophy.

Later could be shown, that the changes in LVMI are closely related to the insulin resistance in animals as well as humans. In an animal experiment comparable could be found the development of structural changes due to insulin resistance in mice within 3 weeks [51]. Verma et al. [52] investigated in 2016 in a subgroup 10 patients with T2DM and performed echo studies at begin and 3 months later. They found a significant reduction in LV mass and an improvement of diastolic function. The mechanism is at all not fully known and needs further elucidation and is not finally to declare at time.

### Influence on insulin resistance

Different compounds and durations of dietary interventions are not comparable in its effects. For this reason the results are not consistent [14, 22].

In this study 27.6% in the verum group and 16.0% in the placebo group showed an euglycaemic IR measured by HOMA-IR at baseline. That suggests that these changes in glucose metabolism could be found in overweight patients much longer before manifestation of T2DM. At study end we found in verum group a reduction in euglycaemic IR to 10.3% and in the placebo group to 8.0%. Only in the verum group these parameters of glucose metabolism were reduced after intervention (*p* = 0.063). The significantly increased serum magnesium concentrations in the verum group compared to the placebo group after intervention were only connected with the significantly decrease of HOMA-IR in the verum group. Chutia and Lynrah [53] showed a positive correlation between fasting insulin level and HOMA level in patients with T2DM compared to non-diabetic controls. The correlation between serum magnesium level and fasting insulin level was inversely.

Low vitamin B12 levels have been found in obese adolescents with clinical features of IR [15]. The supplementation of vitamin B12, folate, and coenzyme Q10 in this study was helpful in two ways: on the one hand in general a great part of the population has a deficiency in these micronutrients, especially seniors [54], and on the other hand the substitution may improve the glucose metabolism with a significant decrease in fasting insulin and HOMA-IR. The BMI remained unchanged throughout the study duration of 6 weeks.

The described effects of coenzyme Q10 on glucose metabolism are differently. In an interventional supplementation of coenzyme Q10 in patients with MetS a positive effect on serum insulin levels and HOMA-IR could be found [21]. In contrary Moazen et al. [20] and Azevedo et al. [5] did not verify an effect of coenzyme Q10 on fasting blood glucose and HbA1C level compared to the placebo group. Other investigators [24] found a significant reduction in fasting plasma glucose and HbA1C level but not in serum insulin and HOMA-IR. The effect on lipid profile was not favourable.

### Influence on other biochemical parameters

The most frequently investigated substance is the coenzyme Q10. Supplementations like vitamin C, E, coenzyme Q10 and selenium only in a combination also improved the glucose and lipid metabolism [22]. Folic acid supplementation in patients with T2DM effects a reduction in homocysteine on the other hand it increases glutathione levels with an inverse effect on HbA1C, TG and HDL-C [14].

In accordance with other authors [21, 24] this study did not show a positive influence on lipids (TC, TG, LDL-C) by CoQ10, which was also given in higher dosages.

CrP was low grade elevated in 20 patients of the verum group and in 16 patients of the placebo group. Even with CrP serum concentrations of> 0.7 mg/l, there is a slightly increased risk [55]. The elevation of CrP indicates in patients with overweight and obesity as well as with metabolic disorders a higher risk for cardiovascular events. In our study was CrP was slightly reduced after dietary supplementation, and elevated in the placebo group. This effect may be due to magnesium intake by dietary supplementation [11]. After administration of coenzyme Q10, h-CrP was also reduced, according to the findings of Raygan et al. [21], and Shargorodski et al. [22] after intake of 60 mg Q10 combined with other antioxidants like vitamin C, E and selenium.

### Influence on cardiac arrhythmias

Magnesium and potassium play an important role in management of CA [9, 12]. CA are often associated with hypomagnesemia but the pathomechanism of this process is not fully understand because of interaction with other electrolyte disturbances [44]. The hypomagnesemia and hypokalemia are closely linked together by triggering of CA [10]. In this pilot study an effect of the dietary supplementation on VPBs, SVPBs and VAS points could not be shown. The reduction of VPBs in the placebo group may be based on a placebo effect. In the verum group we found a significant reduction in heart rate. This may be caused on slowing of sinus node rate by oral magnesium administration [12]. In this pilot study a correlation between changes in LV function and CA could not be found.

### Strength and limitations

The strength of this study includes a sample of patients with cardiovascular disorders like AH, CAD, and frequent PBs, and an averaged overweight, which were treated in an Outpatient Practice for Cardiology. Therefore, all were investigated in one centre and all procedures were under same standardized conditions. Limitations were the relative small number of patients and a post-hoc-analysis. In this echocardiographic study we have not measured routinely all parameters of diastolic function. Since outliers of several parameters are detected on different patients, a general clean-up of the data appears inappropriate, favouring the use of all available data. The tested parameters must be evaluated with other patient’s condition too.

## Conclusion

In this pilot study, dietary intervention with a specific micronutrient combination as add-on to concomitant cardiovascular drug treatment seems to improve cardiometabolic health in patients with CA. The dietary intervention led to a significant decrease in fasting insulin ≥58 pmol/l (*p* = 0.020), and HOMA-IR was reduced in the verum group after 6 weeks (*p* = 0.053). At the same time, parameters of LV diastolic function were improved after intervention in the verum group: significant reduction of LV mass index (*p* = 0.003), and in tendency a decrease of interventricular septal thickness (*p* = 0.053) as well as an increase E/A ratio (*p* = 0.051). The heart rate was significantly decreased (*p* = 0.027), but the effect on reduction of rhythm disturbances like PBs was less pronounced. In both groups serious side effects were not observed. The results of this pilot study give indications for sign of further studies.

## Abbreviations

AH: Arterial hypertension
ALAT: Alanin-aminotransferase
ASAT: Aspartate-aminotransferase
BMI: Body-Mass-Index
BP: Blood Pressure
CA: Cardiac arrhythmias
CAD: Coronary artery disease
Cl: Chloride
CrP: C reactive protein
E/A ratio: Ratio of the early (E) to late (A) ventricular filling velocities
ECG: Electrocardiogram
ES: Effect size
F Insulin: Fasting insulin
FPG: Fasting plasma glucose
GFR: Glomerular filtration rate
HbA1c: Glycated haemoglobin A1c
HDL-C: HDL-cholesterol
Hk: Haematocrit
HOMA-IR: Homeostasis Model Assessment- insulin resistance
IDF: International Diabetes Federation
IGT: Impaired glucose tolerance
IR: Insulin resistance
IVS: Interventricular septum
K: Potassium
LA: Left atrium
LDL-C: LDL-cholesterol
LV: Left ventricular
LVEDD: Left ventricular end diastolic diameter
LVEF: Left ventricular ejection fraction
LVESD: Left ventricular end systolic diameter
LVMI: Left ventricular mass index
LVPW: Left ventricular posterior wall
MCHC: Mean corpuscular/cellular haemoglobin concentration
MCV: Mean corpuscular/cell volume
MetS: Metabolic syndrome
Mg: Magnesium
Na: Natrium
PBs: Premature beats
Q1: First Quartile
Q3: Third Quartile
RVEDD: Right ventricular end diastolic diameter
SV: Supraventricular
SVPBs: Supraventricular premature beats
T2DM: Type 2 diabetes mellitus
TC: Total cholesterol
TG: Triglycerides
VPBs: Ventricular premature beats
WC: Waist Circumference
WHtR: Waist-to-Height-Ratio
